# The Effects of Acute High‐Intensity Interval Exercise and Hyperinsulinemic‐Euglycemic Clamp on Osteoglycin Levels in Young and Middle‐Aged Men

**DOI:** 10.1002/jbm4.10667

**Published:** 2022-09-13

**Authors:** Carlie Bauer, Alexander Tacey, Andrew Garnham, Cassandra Smith, Mary N Woessner, Xuzhu Lin, Navabeh Zarekookandeh, David L Hare, Joshua R Lewis, Lewan Parker, Itamar Levinger

**Affiliations:** ^1^ Institute for Health and Sport Victoria University Melbourne Australia; ^2^ Australian Institute for Musculoskeletal Science Victoria University, University of Melbourne, Western Health St Albans Australia; ^3^ Institute for Nutrition Research, School of Medical and Health Sciences Edith Cowan University Joondalup Australia; ^4^ Medical School The University of Western Australia Perth Australia; ^5^ University of Melbourne and the Department of Cardiology Austin Health Melbourne Australia; ^6^ University of Western Australia and Centre for Kidney Research Children's Hospital at Westmead Westmead Australia; ^7^ School of Public Health Sydney Medical School, The University of Sydney Sydney Australia; ^8^ Institute for Physical Activity and Nutrition (IPAN), School of Exercise and Nutrition Sciences Deakin University Geelong Australia

**Keywords:** AGING, BONE‐MUSCLE INTERACTIONS, EXERCISE, HUMAN ASSOCIATION STUDIES, METABOLISM

## Abstract

Osteoglycin (OGN) is a leucine‐rich proteoglycan that has been implicated in the regulation of glucose in animal models. However, its relationship with glucose control in humans is unclear. We examined the effect of high‐intensity interval exercise (HIIE) and hyperinsulinemic‐euglycemic clamp on circulating levels of OGN as well as whether circulating OGN levels are associated with markers of glycemic control and cardio‐metabolic health. Serum was analyzed for OGN (ELISA) levels from 9 middle‐aged obese men (58.1 ± 2.2 years, body mass index [BMI] = 33.1 ± 1.4 kg∙m^−2^, mean ± SEM) and 9 young men (27.8 ± 1.6 years, BMI = 24.4 ± 0.08 kg∙m^−2^) who previously completed a study involving a euglycemic‐hyperinsulinemic clamp at rest and after HIIE (4x4 minutes cycling at approximately 95% peak heart rate (HRpeak), interspersed with 2 minutes of active recovery). Blood pressure, body composition (dual‐energy X‐ray absorptiometry), and insulin sensitivity (hyperinsulinemic‐euglycemic clamp) were assessed. Serum OGN was higher in the young cohort compared with the middle‐aged cohort (65.2 ± 10.1 ng/mL versus 36.5 ± 4. 5 ng/mL, *p* ≤ 0.05). Serum OGN was unaffected by acute HIIE but decreased after the insulin clamp compared with baseline (~−27%, *p* = 0.01), post‐exercise (~−35%, *p* = 0.01), and pre‐clamp (~−32%, *p* = 0.02) time points, irrespective of age. At baseline, lower circulating OGN levels were associated with increased age, BMI, and fat mass, whereas higher OGN levels were related to lower fasting glucose. Higher OGN levels were associated with a higher glucose infusion rate. Exercise had a limited effect on circulating OGN. The mechanisms by which OGN affects glucose regulation should be explored in the future. © 2022 The Authors. *JBMR Plus* published by Wiley Periodicals LLC on behalf of American Society for Bone and Mineral Research.

## 1 Introduction

Overweight and obesity have reached epidemic levels with more than 39% of the global population classified as being overweight and an additional 13% classified as obese.^(^ ^1^ ^)^ Excess adiposity can have major health implications and is a risk factor for several comorbidities including insulin resistance and type 2 diabetes.^(^ ^2^, ^3^ ^)^ Aging is known to coincide with a decline in cardio‐metabolic health, including decreased insulin sensitivity and impaired glycemic control, further contributing to the risk of developing cardio‐metabolic disease.^(^ ^4^ ^)^ Exercise, in particular high‐intensity interval exercise (HIIE), has a profound positive effect on insulin sensitivity,^(^ ^5^ ^)^ yet the mechanisms involved in the insulin sensitizing effects of exercise are not fully understood.^(^ ^5^, ^6^ ^)^ In addition, HIIE has been suggested to be superior compared with moderate‐intensity exercise in improving glucose regulation.^(^ ^7^, ^8^ ^)^

Research has identified the important role of bone and muscle cross‐talk in regulating energy homeostasis and glucose metabolism.^(^ ^9^, ^10^ ^)^ As such, there has been increased interest surrounding the identification of hormones secreted by both bone and muscle that may interact to mediate glucose regulation. Several previous studies have supported a potential link between circulating osteocalcin (OC), an osteoblast‐specific hormone, and glucose regulation.^(^ ^11^, ^12^, ^13^, ^14^, ^15^ ^)^ However, bone and muscle cross‐talk is likely to be multifactorial, and thus there has been ongoing interest in identifying other novel biomarkers that may mediate glucose regulation.^(^ ^16^, ^17^ ^)^

Osteoglycin (OGN), a leucine‐rich proteoglycan released by bone, muscle, and other tissues, has been implicated in glucose regulation independent of OC.^(^ ^18^ ^)^ Animal studies have demonstrated that OGN has an active role in glucose homeostasis.^(^ ^18^ ^)^ Administration of OGN to OGN‐deficient mice 4 hours before an insulin tolerance test improves insulin action, suggesting OGN is involved in metabolic pathways responsible for insulin regulation.^(^ ^18^ ^)^ Despite the positive results in animal models, research connecting OGN to glucose metabolism in humans is limited and conflicting.^(^ ^18^ ^)^ Elevated serum OGN in severely obese men and women was correlated with lower circulating blood glucose after bariatric surgery.^(^ ^18^ ^)^ In contrast, others have reported that obese individuals have increased levels of OGN compared with non‐obese individuals, although OGN levels were measured specifically within adipose tissue.^(^ ^19^ ^)^ As such, the role of circulating OGN in humans and its influence on energy homeostasis remains unclear.

It is possible that OGN is mediated by acute exercise. We previously showed that acute HIIE leads to a significant increase in post‐exercise insulin sensitivity, which correlates with increased circulating OC in young^(^ ^13^ ^)^ and middle‐aged men.^(^ ^11^ ^)^ Given the likely role of OGN in energy homeostasis and the influence of acute exercise on other bone‐derived hormones, it is possible that OGN may be implicated in the beneficial effects of exercise on insulin sensitivity. However, to our knowledge no study has investigated the effects of acute exercise or insulin stimulation on OGN in young healthy adults or middle‐aged obese individuals.

The primary aims of this study were to examine the effect of HIIE and a hyperinsulinemic‐euglycemic clamp on circulating levels of OGN. We also examined whether circulating levels of OGN are associated with markers of glycemic control and cardio‐metabolic health.

## 2 Subjects and Methods

This is a secondary analysis of serum samples collected from middle‐aged obese males^(^ ^11^ ^)^ and young healthy males^(^ ^13^ ^)^ who underwent an acute HIIE session followed by a 2‐hour euglycemic hyperinsulinemic clamp (insulin clamp).

### 2.1 Participants

Data from 11 middle‐aged obese men (58.1 ± 2.2 years, body mass index [BMI] = 33.1 ± 1.4 kg∙m^−2^, mean ± SEM) and 9 young men (27.8 ± 1.6 years, BMI = 24.4 ± 0.08 kg∙m^−2^) was included in this study. Exclusion criteria included bone disease, musculoskeletal or other conditions affecting the capacity to perform activities of daily living, uncontrolled or symptomatic metabolic or cardiovascular disease, and use of medications such as warfarin, vitamin K supplementation or anti‐hyperglycemic medications affecting insulin secretion, insulin sensitivity, or bone metabolism. All participants signed an informed consent following written and verbal explanation of study details. The study was approved by the Victoria University Human Research Ethics Committee.

### 2.2 Study overview

Circulating OGN was analyzed in serum samples collected from two previously published studies.^(^ ^11^, ^13^ ^)^ Because of the limited amount of serum, OGN could only be analyzed for 10 of the 11 people in the middle‐aged group at each time point. In brief, participants completed an initial screening visit, which included anthropometric measures and a graded exercise test to determine peak aerobic capacity (VO_2peak_). Fasting insulin, glucose, glycosylated hemoglobin (HbA1c), triglyceride, and high‐density lipoprotein (HDL) were assessed at Austin Health (Melbourne, Australia) pathology using standard hospital assay protocols. Height was measured using a standard stadiometer and body weight was measured with a scale (TANITA, Tanita Corporation, Tokyo, Japan). Blood pressure was measured at rest with participants seated for at least 15 minutes using a standard mercury sphygmomanometer.

### 2.3 Graded exercise test

A signs and symptom‐limited graded exercise test on a cycle ergometer was completed to assess VO_2peak_ and peak heart rate (HR_peak_), as previously reported.^(^ ^11^, ^13^ ^)^

### 2.4 Experimental session (acute HIIE and insulin clamp)

For the experimental session, participants attended the laboratory at approximately 8 a.m. after an overnight fast. An intravenous cannula was inserted into an antecubital fossa vein of both arms (one for blood sampling and one for intravenous infusion of insulin and glucose). After approximately 30 minutes of rest on a hospital bed, a baseline blood sample was collected and then participants completed a single session of HIIE. Immediately after exercise, the young and healthy males rested for 3 hours on a bed and then underwent a 2‐hour insulin clamp,^(^ ^11^ ^)^ whereas the middle‐aged obese males rested for 1 hour on a bed before undergoing the 2‐hour insulin clamp.^(^ ^13^ ^)^ Venous blood samples were collected at baseline (before exercise), immediately after exercise, before the insulin clamp, and after the insulin clamp.

### 2.5 Acute HIIE

The same cycling protocol was used for the young individuals and middle‐aged individuals. This included a 4‐ to 6‐minute warm‐up at an intensity of approximately 50% to 60% of HR_peak_, followed by four sets of 4‐minute cycling intervals at 90% to 95% HR_peak_, which were interspersed with 2‐minute active recovery periods at 50% to 60% HR_peak_. The workload (watts) was adjusted during the session to maintain the target heart rate intensity.

### 2.6 Post‐exercise insulin clamp

Insulin (Actrapid; Novo Nordisk, Bagsvaerd, Denmark) was infused for 120 minutes at a rate of 40 mU.m^−2^ per minute to produce a stable and elevated insulin concentration in the final 30 minutes of the insulin clamp. The glucose infusion rate (GIR; mg·kg^−1^·min^−1^) and the GIR per unit of insulin (M‐Value) were assessed in the final 30‐minute insulin‐stimulated period to determine insulin sensitivity.^(^ ^20^ ^)^ Exogenous glucose was infused with a standard syringe pump (TE‐311, Terumo, Japan) during the insulin clamp using a variable infusion rate to maintain a blood glucose level target of approximately 5 mmol/L. Blood glucose levels were assessed every 5 minutes using an automated analyzer (YSI 2300 STAT Plus Glucose & Lactate Analyzer, Yellow Springs, United States) and arterialized blood obtained using an arm warmer set to approximately 50°C.

### 2.7 Serum OGN analysis

Venous blood was collected using SST collection tubes and allowed to clot at room temperature for 15 minutes. Samples were then centrifuged (10 minutes at 2000 x g, 4°C) and the serum immediately aliquoted and stored at −80°C until analyzed. Serum OGN was analyzed using an ELISA (Cloud‐Clone Corp, Houston, TX, USA) according to the manufacturer protocol. To obtain an optimal dilution factor for the sample conditions, a series of sample dilutions were first conducted. After optimization, serum samples were diluted 1:80 with standard diluent for the young individuals and 1:60 for the middle‐aged individuals. This ELISA has been used in previous studies.^(^ ^18^ ^)^

### 2.8 Statistical analysis

Data were checked for normality and analyzed using Prism statistical analysis software (GraphPad Prism 9.2.0, GraphPad, La Jolla, CA, USA). Non‐normally distributed data were log transformed using the natural log to approximate normal distribution before statistical analysis. Comparisons of multiple means were analyzed using a two‐factor mixed model analysis of variance (ANOVA) with time (baseline, post‐exercise, pre‐clamp, and post‐clamp) as a within‐subjects factor and age (young and middle‐aged adults) as a between‐subjects factor. Significant interaction and main effects were explored post hoc with Fisher's least significant difference test. Unpaired *t* tests were conducted to examine the differences in baseline participant characteristics between the young and middle‐aged cohorts. Linear regressions were conducted to analyze the association of OGN with physiological characteristics and glycemic control variables at baseline and post‐exercise. The level of significance was set at 95% (*p* ≤ 0.05) for statistical analyses. Data are reported as mean ± standard error of mean (SEM).

## 3 Results

### 3.1 Baseline

Participant characteristics are presented in Table 1. The middle‐aged males were older and had higher BMI, fasting glucose, fasting insulin and HbA1c, and lower OGN and VO_2peak_ compared with the young males (*p* < 0.05).

**Table 1:** Participant Characteristics

Variables	Young (*n* = 9)	Middle‐aged (*n* = 11)
Age (years)	27.8 ± 1.6	58.1 ± 2.1***
BMI (kg/m^2^)	24.4 ± 0.1	33.1 ± 1.4***
Weight (kg)	80.0 ± 3.8	102.5 ± 3.9***
Height (cm)	180.6 ± 3.1	176.2 ± 1.7
Body fat (%)	18.2 ± 1.7	36.4 ± 1.5***
VO_2peak_ (mL/kg/min)	46.1 ± 3.2	21.9 ± 1.6***
OGN (ng/mL)	65.2 ± 10.1	36.5 ± 4.5*, ^a^
Fasting glucose (mmol/L)	4.4 ± 0.1	5.3 ± 0.2**
Fasting insulin (μU/mL)	6.5 ± 0.8	12.7 ± 2.5*
HbA1c (%)	5.2 ± 0.1	5.6 ± 0.1***

BMI = body mass index; VO_2peak_ = peak aerobic capacity; OGN = osteoglycin; HbA1c = glycosylated hemoglobin.

Values are mean ± SEM.

* *p* ≤ 0.05.

** *p* ≤ 0.01.

*** *p* ≤ 0.001 between young and middle‐aged individuals.

^a^ *n* = 10 for OGN in the middle‐aged cohort.

**Fig. 1:**
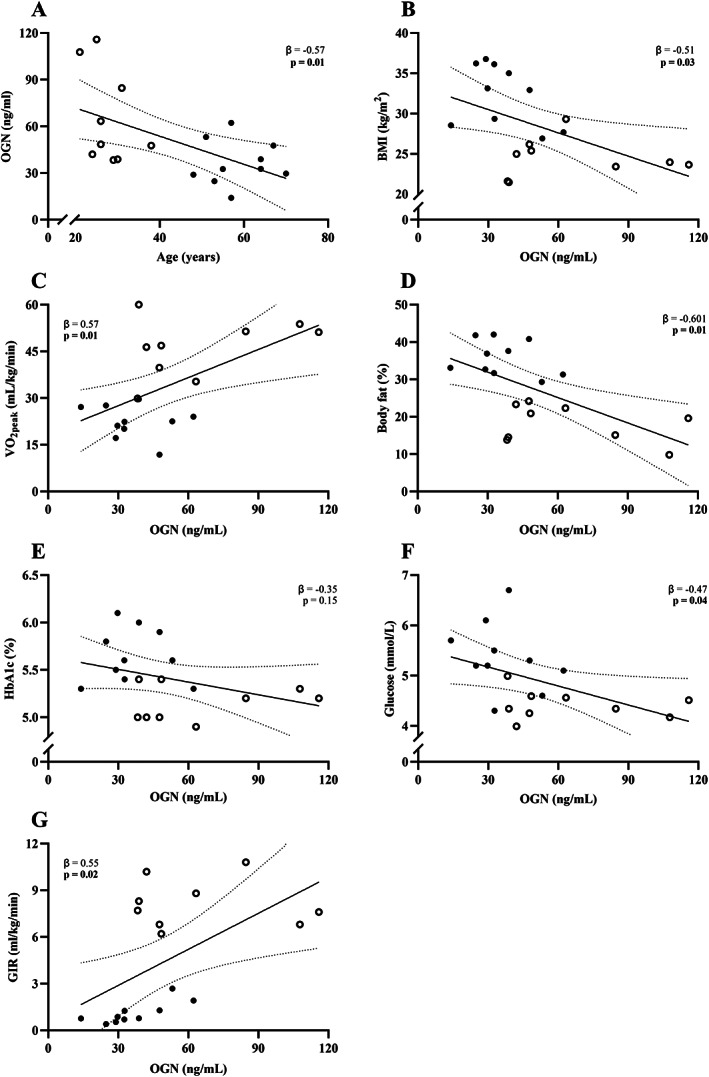
Association of baseline OGN with (*A*) BMI, (*B*) age, (*C*) VO_2_, (*D*) body fat, (*E*) HBA1c, and (*F*) fasting glucose at baseline (*G*) GIR. ○ indicates young men (*n* = 9); ● indicates middle‐aged men (*n* = 10). Values are mean ± SEM; bold indicates *p* < 0.05. OGN = osteoglycin; HBA1c = glycosylated hemoglobin; BMI = body mass index; GIR = glucose infusion rate.

At baseline, linear regressions using the pooled data revealed that higher OGN was associated with lower BMI, age, percent body fat and fasting glucose, and a higher VO_2peak_ (Fig. 1, *p* < 0.05 for all). Age explains 32% of the variation in OGN levels. For every 1 year increase in age, there is a decrease in OGN levels of 0.57 ng/mL (*p* = 0.01) (Fig. 1*A*). OGN explains 26% of the variation in BMI. For every 1 ng/mL increase in OGN, there is a 0.51 kg/m^2^ decrease in BMI (*p* = 0.03) (Fig. 1*B*). Higher OGN was associated with higher insulin sensitivity as measured by GIR (*p* = 0.03). Baseline OGN accounts for 30% of the variation with GIR. With every 1 ng/mL increase in OGN, there was a 0.55 unit increase in GIR (*p* = 0.02) (Fig. 1*G*).

### 3.2 Effect of acute exercise and insulin clamp on OGN

**Fig. 2:**
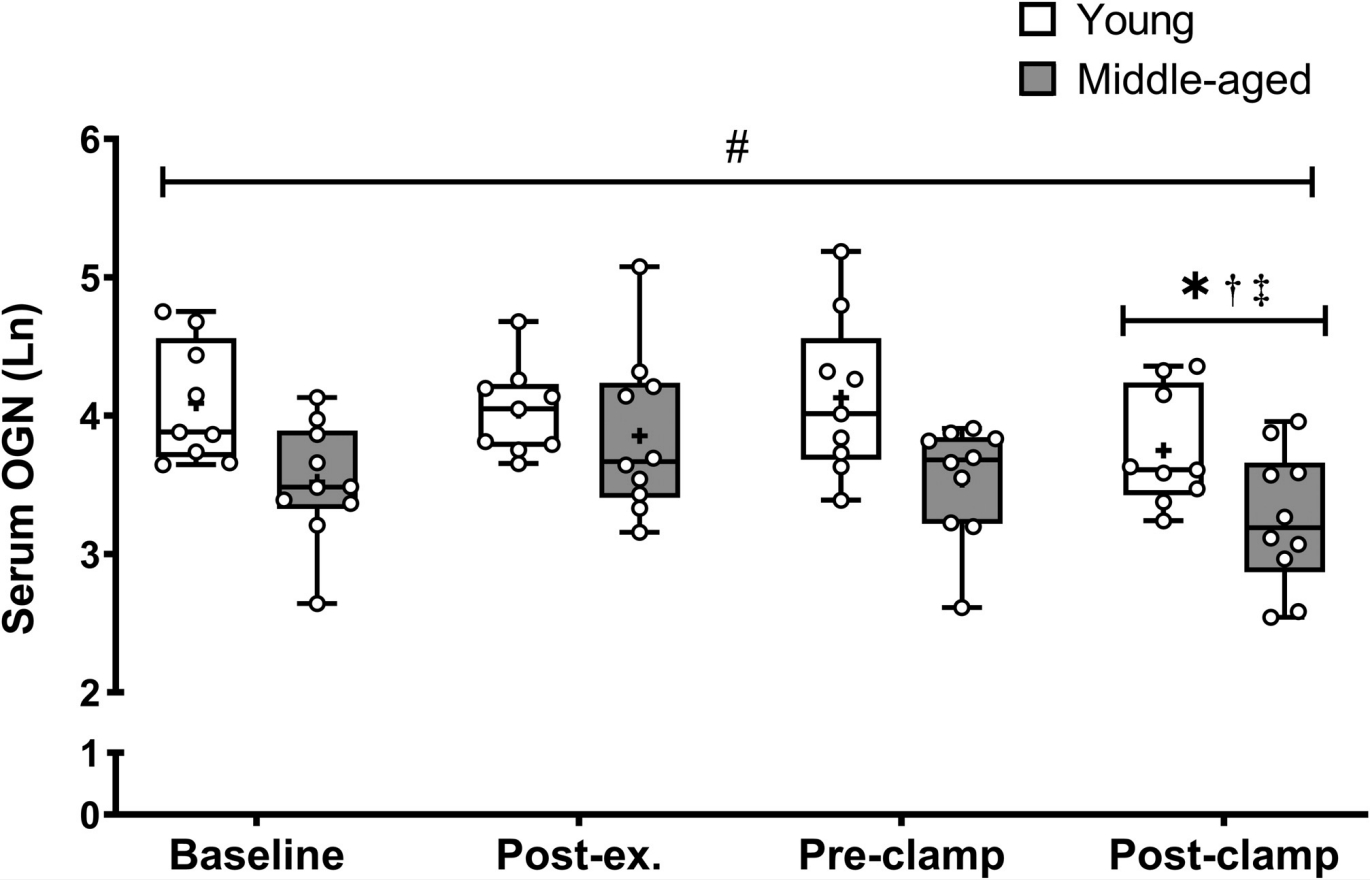
Serum OGN after high‐intensity interval exercise and an insulin clamp in young (*n* = 9) and middle‐aged adults (*n* = 10 at each time point). #*p* < 0.01 main effect for condition. *p < 0.01 compared with baseline. †*p* < 0.001 compared with post‐exercise. ‡*p* < 0.01 compared with pre‐clamp. Data are presented as a box and whisker plot. The box represents the interquartile range alongside the median (line) and mean (plus symbol). The whiskers represent the minimum and maximum range of the data.

There was no interaction effect between time and age for serum OGN (*p* = 0.27). Main effects for age (*p* = 0.01) and time (*p* < 0.01) were detected (Fig. 2). Serum OGN was higher in the young cohort (by ~55%) compared with the middle‐aged cohort, irrespective of time point. Serum OGN was lower post insulin clamp compared with baseline (~−27%, *p* = 0.01), post‐exercise (~−35%, *p* = 0.01), and pre‐clamp time points (~−32%, *p* = 0.02), irrespective of age.

#### 3.2.1 Associations post‐exercise

No significant association between OGN and glucose levels were observed post‐exercise in the pooled analysis (*p* > 0.05 for all, data not shown).

## 4 Discussion

We report that middle‐aged obese males have lower circulating levels of serum OGN compared with young individuals. Despite no changes in OGN levels post‐acute HIIE, insulin stimulation after exercise via a hyperinsulinemic‐euglycemic clamp led to decreased OGN levels in the study cohort. We also report that at baseline, lower circulating OGN levels are associated with increased age, BMI, and fat mass. In addition, higher OGN was associated with lower fasting glucose at baseline. Higher OGN was associated with higher insulin sensitivity as assessed by higher GIR. This finding supports the hypothesis that OGN may play a mediating role in energy homeostasis and cardio‐metabolic health in humans.

It is now recognized that the secretion of regulatory factors from bone and muscle affect each other, forming a dynamic and complex model of tissue cross‐talk that is crucial for glucose regulation.^(^ ^10^ ^)^ Although factors involved in this cross‐talk are not well established, emerging research suggests OGN is a potential mediator of glucose regulation and insulin sensitivity.^(^ ^17^, ^18^ ^)^ In support of this, we reported that lower OGN levels are associated with higher fasting glucose levels. We detected an association between baseline OGN and insulin sensitivity as measured by GIR. No changes in circulating levels of OGN after exercise were observed. Because muscle is the largest site for glucose disposal in response to insulin,^(^ ^21^ ^)^ it is possible that local changes in OGN within skeletal muscle were not identified and should be explored in future studies. Furthermore, it was previously reported that insulin sensitivity after exercise was associated with increased undercarboxylated osteocalcin, another hormone implicated in bone‐muscle cross‐talk.^(^ ^11^ ^)^ Mechanistic studies suggest that OGN has a direct effect on insulin sensitivity and glucose metabolism independent of OC, though this was in an animal model as compared to a human model in the current study.^(^ ^18^ ^)^ As far as we are aware, this is the first study investigating the relationship between exercise, OGN, and insulin sensitivity in humans. Although previous research is yet to adequately investigate OGN changes after exercise, a study in OGN‐deficient mice showed significantly decreased physical activity over a 3‐day period.^(^ ^18^ ^)^ Combined with our finding that higher OGN levels were associated with higher VO_2peak_ levels, it is possible that serum OGN is involved in exercise capacity and post‐exercise insulin sensitivity.

Aging is known to have a profound negative effect on human physiology, including bone and muscle. Changes in circulating hormones are implicated in these processes.^(^ ^22^ ^)^ We report that increased age is associated with lower OGN levels. This is consistent with previous findings in mice models that OGN is downregulated with age.^(^ ^23^ ^)^ It is possible that the higher OGN levels in younger people are due to increased bone formation and higher muscle mass, which would both require increased energy use,^(^ ^24^ ^)^ though this is beyond the scope of the current study and would be worthwhile to explore in future studies.

There is a known relationship between increased BMI and decreased insulin sensitivity, though not all obese people are insulin‐resistant.^(^ ^25^, ^26^, ^27^, ^28^ ^)^ It has been hypothesized that OGN has an endocrine function in glucose metabolism; however, this was based on evidence primarily observed in animal studies. Previous research suggests greater reductions in BMI are associated with greater increases in OGN levels post weight loss, which is associated with improved blood glucose control.^(^ ^18^ ^)^ This is consistent with findings of the current study that lower BMI is associated with increased OGN at baseline.

The post hoc design of the study as well as the relatively small sample size are potential limitations of this study. Invasive studies typically have small sample sizes; however, we did find significant preliminary outcomes linking changes in OGN to changes in insulin sensitivity after the insulin clamp. This study combined cohorts from two separate studies with slight variations in protocols. The acute exercise protocol and insulin clamps procedures were identical, yet the clamp in the middle‐aged study started 1 hour post‐exercise compared with 3 hours post‐exercise in the younger cohort. Further investigation of OGN and glucose metabolism in humans using gold standard techniques such as the insulin clamp and glucose tracers is warranted.

In conclusion, we show that middle‐aged obese individuals have lower circulating OGN compared with young, lean, and more insulin‐sensitive adults. This may indicate that serum OGN is related to the increased cardio‐metabolic risk observed with aging and excess adiposity, a notion supported by the findings that age, BMI, and fat mass were associated with lower circulating OGN levels in the pooled data. Though further studies are needed to determine if this is a cause‐and‐effect relationship, the findings of the current study may be hypothesis generating for future research. Although acute exercise did not directly influence serum OGN, higher serum OGN levels were associated with greater post‐exercise insulin sensitivity. Future studies should explore whether OGN has a direct effect on muscle metabolism and the mechanisms involved.

## Disclosures

All authors state that they have no conflicts of interest.

## Author Contributions

**Carlie Bauer:** Conceptualization; data curation; formal analysis; methodology; writing – original draft; writing – review and editing. **Alexander Tacey:** Conceptualization; formal analysis; methodology; writing – original draft; writing – review and editing. **Andrew Garnham:** Investigation; writing – review and editing. **Cassandra Smith:** Formal analysis; writing – review and editing. **Mary N Woessner:** Formal analysis; writing – review and editing. **Xuzhu Lin:** Formal analysis; investigation; methodology; writing – review and editing. **Navabeh Zarekookandeh:** Formal analysis; investigation; methodology; writing – review and editing. **David L Hare:** Conceptualization; methodology; project administration; writing – review and editing. **Joshua R Lewis:** Formal analysis; writing – review and editing. **Lewan Parker:** Conceptualization; formal analysis; investigation; methodology; project administration; supervision; writing – original draft; writing – review and editing. **Itamar Levinger:** Conceptualization; investigation; methodology; project administration; supervision; writing – original draft; writing – review and editing.

### Peer Review

The peer review history for this article is available at https://publons.com/publon/10.1002/jbm4.10667.

## Data Availability

The data that support the findings of this study are available from the corresponding author upon reasonable request and approval of Victoria University Human Ethics Committee.
